# PPP6C Negatively Regulates STING-Dependent Innate Immune Responses

**DOI:** 10.1128/mBio.01728-20

**Published:** 2020-08-04

**Authors:** Guoxin Ni, Zhe Ma, Jason P. Wong, Zhigang Zhang, Emily Cousins, M. Ben Major, Blossom Damania

**Affiliations:** a Department of Microbiology and Immunology, University of North Carolina at Chapel Hill, Chapel Hill, North Carolina, USA; b Lineberger Comprehensive Cancer Center, University of North Carolina at Chapel Hill, Chapel Hill, North Carolina, USA; University of Pittsburgh School of Medicine

**Keywords:** PPP6C, STING, phosphorylation, phosphatase, interferon, KSHV, HSV-1, VSV, Kaposi's sarcoma-associated herpesvirus, PP6C, herpes simplex virus, interferons, protein phosphorylation, vesicular stomatitis virus

## Abstract

Stimulator of interferon genes (STING) is an essential adaptor protein of the innate DNA-sensing signaling pathway, which recognizes genomic DNA from invading pathogens to establish antiviral responses in host cells. STING activity is tightly regulated by several posttranslational modifications, including phosphorylation. However, specifically how the phosphorylation status of STING is modulated by kinases and phosphatases remains to be fully elucidated. In this study, we identified protein phosphatase 6 catalytic subunit (PPP6C) as a binding partner of Kaposi’s sarcoma-associated herpesvirus (KSHV) open reading frame 48 (ORF48), which is a negative regulator of the cyclic GMP-AMP synthase (cGAS)-STING pathway. PPP6C depletion enhances double-stranded DNA (dsDNA)-induced and 5′ppp double-stranded RNA (dsRNA)-induced but not poly(I:C)-induced innate immune responses. PPP6C negatively regulates dsDNA-induced IRF3 activation but not NF-κB activation. Deficiency of PPP6C greatly inhibits the replication of herpes simplex virus 1 (HSV-1) and vesicular stomatitis virus (VSV) as well as the reactivation of KSHV, due to increased type I interferon production. We further demonstrated that PPP6C interacts with STING and that loss of PPP6C enhances STING phosphorylation. These data demonstrate the important role of PPP6C in regulating STING phosphorylation and activation, which provides an additional mechanism by which the host responds to viral infection.

## Abstract (precis)

Cytosolic DNA, which usually comes from invading microbes, is a dangerous signal to the host. The cGAS-STING pathway is the major player that detects cytosolic DNA and then evokes the innate immune response. As an adaptor protein, STING plays a central role in controlling activation of the cGAS-STING pathway. Although transient activation of STING is essential to trigger the host defense during pathogen invasion, chronic STING activation has been shown to be associated with several autoinflammatory diseases. Here, we report that PPP6C negatively regulates the cGAS-STING pathway by removing STING phosphorylation, which is required for its activation. Dephosphorylation of STING by PPP6C helps prevent the sustained production of STING-dependent cytokines, which would otherwise lead to severe autoimmune disorders. This work provides additional mechanisms on the regulation of STING activity and might facilitate the development of novel therapeutics designed to prevent a variety of autoinflammatory disorders.

## INTRODUCTION

Host cells have evolved a variety of germ line-encoded pattern recognition receptors to detect structurally conserved components named pathogen-associated molecular patterns (PAMPs) from invading microbes, which upon detection by the cell triggers a cascade of signal transduction, leading to eventual establishment of an antiviral immune response (1). Microbial genomic RNA and DNA present in the cytoplasm are major PAMPs which are predominantly recognized by retinoic acid-inducible gene I protein (RIG-I)-like receptors (RLRs) and the cyclic GMP-AMP synthase (cGAS)-stimulator of interferon gene (STING) pathways (2). Cytosolic RNA is mainly detected by RIG-I and melanoma differentiation-associated gene 5 (MDA5), which then signals through the adaptor protein mitochondrial antiviral signaling (MAVS; also known as Cardif, VISA, and IPS-1) to activate downstream signaling (2). Microbial DNA or inappropriately processed self-DNA in the cytosol is sensed by cGAS, which generates a small second messenger 2′3′-cyclic GMP-AMP (cGAMP) upon binding of DNA (3–6). cGAMP interacts and activates the endoplasmic reticulum (ER)-resident adaptor protein STING (also known as MITA and ERIS), which then translocates from the ER via the Golgi apparatus to the perinuclear region (7–10). Activation of both MAVS and STING leads to phosphorylation of transcription factors such as interferon regulatory factor 3 (IRF3) and nuclear factor kappa B (NF-κB) (2, 11). Activated IRF3 and NF-κB translocate to the nucleus to initiate the transcription of numerous innate immune genes, including type I interferons (IFNs) and proinflammatory cytokines.

Although transient activation of the cGAS-STING pathway is essential to trigger the host defense in response to pathogen invasion, chronic STING activation has been shown to be associated with several autoinflammatory diseases, such as Aicardi-Goutieres syndrome (AGS), systemic lupus erythematosus (SLE), and STING-associated vasculopathy with onset in infancy (SAVI) (12–14), as well as inflammation-driven tumor genesis (15). Therefore, tight control of STING activity is required to prevent sustained production of cytokines, which would otherwise lead to autoimmune diseases. Several posttranslational modifications have been shown to delicately regulate STING activity, such as phosphorylation, ubiquitination, and palmitoylation (11, 16–18). During the translocation from the ER to the perinuclear region, STING is phosphorylated by TANK-binding kinase 1 (TBK1) at serine 366, which is required for IRF3 recruitment to the STING-TBK1 complex (16). However, another study indicated that STING phosphorylation by unc-51-like autophagy-activating kinase 1 (ULK1) at the same site negatively regulates STING activity (11). Therefore, the role of phosphorylation in modulating STING activity still awaits further characterization. The protein phosphatase Mg^2+^/Mn^2+^-dependent 1A (PPM1A) has been shown to antagonize TBK1-mediated STING phosphorylation (19). Whether there are other phosphatases involved in regulating STING phosphorylation remains an open question.

Protein phosphatase 6 (PP6) is a member of the phosphoprotein phosphatase (PPP) family of Ser/Thr protein phosphatases, which are conserved throughout eukaryotes (20). PP6 works as a holoenzyme that contains the catalytic subunit PPP6C and some of the seven known regulatory subunits (PPP6R1, PPP6R2, PPP6R3, ankyrin repeat domain 28 [ANKRD28], ANKRD44, ANKRD52 and alpha 4). PP6 exhibits multiple roles in regulating several cellular processes, including cell cycle, autophagy, DNA damage repair and lymphocyte development (20). Mutations in PPP6C have been linked to melanoma carcinogenesis (21, 22). PPP6C negatively regulates NF-κB signaling by stabilizing NF-κB inhibitor ε (IκBε) and suppressing sustained mitogen-activated protein kinase 7 (MAP3K7/TAK1) activation (23, 24). In this study, we report that PPP6C negatively regulates RIG-I-dependent and STING-dependent innate immune responses. Depletion of PPP6C greatly inhibits herpes simplex virus 1 (HSV-1) and vesicular stomatitis virus (VSV) replication as well as Kaposi’s sarcoma-associated herpesvirus (KSHV) reactivation. We further demonstrate that PPP6C modulates STING activity by interacting with and dephosphorylating STING.

## RESULTS

### PPP6C interacts with KSHV ORF48.

A previous study in our lab identified KSHV ORF48 protein as a potential negative regulator of the cGAS-STING pathway in a screen of KSHV open reading frames (ORFs) involved in down-modulating this pathway (25). Overexpression of ORF48 in HEK293T cells inhibited cGAS-STING-induced IFN-β–luciferase activity in this screen (25). To identify potential host proteins that interact with ORF48, we established EA.hy926 cell lines that stably express control empty vector (EV) or FLAG-tagged ORF48. Immunoprecipitation assays using anti-FLAG affinity beads were performed with these two cell lines, and samples were subjected to mass-spectrometric analysis. Proteins that show increased abundance in samples precipitated from cells expressing ORF48 compared to control cells were considered potential ORF48-interacting partners. The top 10 hits are listed in Fig. S1A in the supplemental material, with ORF48 itself as the first hit. Five of the 10 proteins come from the protein phosphatase 6 (PP6) complex, including the catalytic subunit PPP6C and four regulatory subunits: PPP6R1, PPP6R2, PPP6R3, and ankyrin repeat domain 28 (ANKRD28) (Fig. S1A). To confirm the interaction between ORF48 and PPP6C, a similar immunoprecipitation was performed as described above with the two stable cell lines, and immunoblotting showed that FLAG-tagged ORF48 indeed interacts with endogenous PPP6C in EA.hy926 cells (Fig. S1B). HEK293T cells were transfected with HA-tagged PPP6C or streptavidin (Strep)-tagged ORF48 individually or together. Cells were harvested and subjected to a coimmunoprecipitation assay, which further confirmed that PPP6C interacts with ORF48 (Fig. S1C and D).

10.1128/mBio.01728-20.1 FIG S1 PPP6C interacts with KSHV ORF48. (A) EA.hy926 cells that stably express control empty vector or FLAG-tagged KSHV ORF48 were immunoprecipitated with anti-FLAG beads and subjected to mass-spectrometric analysis. The top 10 proteins that showed the greatest increase in abundance in the ORF48-expressing cells compared to control cells are listed. (B) The immunoprecipitated samples from panel A were immunoblotted with the indicated antibodies. (C and D) HA-tagged PPP6C and Strep-tagged ORF48 were transfected individually or together into HEK293T cells for 30 h. Cell lysates were immunoprecipitated with anti-HA antibody (C) or streptavidin beads (D) and then immunoblotted with the indicated antibodies. The data in panels B to D are representative of two independent experiments. Download FIG S1, PDF file, 1.2 MB. Copyright © 2020 Ni et al. 2020 Ni et al. This content is distributed under the terms of the Creative Commons Attribution 4.0 International license.

### PPP6C depletion enhances 5′ppp dsRNA- and dsDNA-induced but not poly(I:C)-induced innate immune responses.

**FIG 1:**
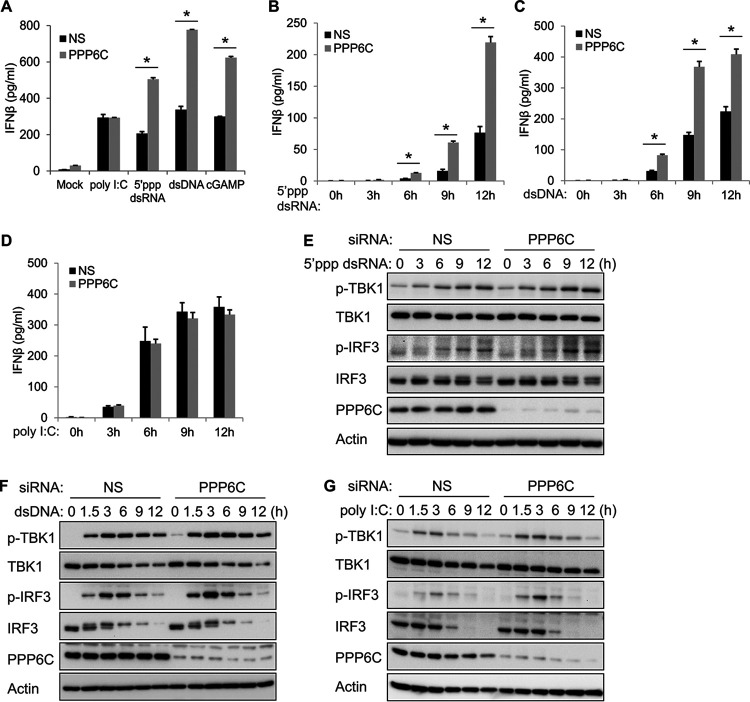
PPP6C depletion enhances 5′ppp dsRNA- and dsDNA-induced but not poly(I:C)-induced innate immune responses. (A) EA.hy926 cells were transfected with control nonspecific (NS) or PPP6C siRNA for 72 h and then transfected with poly(I:C) (0.5 μg/ml), 5′ppp dsRNA (4 μg/ml), dsDNA (4 μg/ml), or cGAMP (4 μg/ml) for 16 h. IFN-β production in the supernatant was measured by ELISA. (B to D) EA.hy926 cells were transfected with NS or PPP6C siRNA for 72 h and then transfected with 5′ppp dsRNA (B), dsDNA (C), or poly(I:C) (D) for the indicated time periods. IFN-β production in the supernatant was measured by ELISA. (E to G) EA.hy926 cells were transfected with NS or PPP6C siRNA for 72 h and then transfected with 5′ppp dsRNA (E), dsDNA (F), or poly(I:C) (G) for the indicated times. Cell lysates were immunoblotted with the indicated antibodies. The data shown are representative of three independent experiments. Data in panels A to D are presented as mean + SD. *, *P* < 0.05 by Student's *t* test.

Considering that ORF48 is a negative regulator of the cGAS-STING pathway (25) and PPP6C interacts with ORF48, we further evaluated whether PPP6C is also involved in regulating the innate immune response. After transfection of control nonspecific (NS) or PPP6C small interfering RNA (siRNA) for 72 h, EA.hy926 cells were transfected with 5′ppp double-stranded RNA (dsRNA) or poly(I:C) (which are ligands of RIG-I and MDA5, respectively, and activate the RNA sensing pathway) or transfected with dsDNA or cGAMP (which are ligands of cGAS and STING, respectively, and activate the DNA sensing pathway), for 16 h. Knockdown of PPP6C significantly increased 5′ppp dsRNA-, dsDNA-, and cGAMP-induced IFN-β production compared to control siRNA-transfected cells, while poly(I:C)-induced IFN-β production was largely unaffected in PPP6C-depleted cells (Fig. 1A). Consistently, knockdown of PPP6C markedly enhanced IFN-β production induced by 5′ppp dsRNA or dsDNA at various time points after transfection (Fig. 1B and C) but did not affect poly(I:C)-induced IFN-β production (Fig. 1D). Activation of either the RLR pathway or the cGAS-STING pathway leads to phosphorylation of TBK1 and IRF3, which then activates transcription of type I IFN and proinflammatory genes (2). While phosphorylation of TBK1 in PPP6C-depleted cells was largely unaffected or only slightly increased after transfection of 5′ppp dsRNA or dsDNA, phosphorylation of IRF3 was greatly increased in PPP6C-deficient cells in response to 5′ppp dsRNA or dsDNA stimulation (Fig. 1E and F). Consistent with the enzyme-linked immunosorbent assay (ELISA) data (Fig. 1D), knockdown of PPP6C did not significantly affect poly(I:C)-induced IRF3 phosphorylation, although we did see slightly increased IRF3 phosphorylation at 3 h poststimulation (Fig. 1G). The knockdown efficiency of PPP6C in all experiments was confirmed by immunoblotting (Fig. 1E to G).

In order to rule out the nonspecific effect of the pooled PPP6C siRNA, we also tested three different individual PPP6C siRNAs. All three siRNAs efficiently suppressed PPP6C expression (Fig. S2A). 5′ppp dsRNA- or dsDNA-induced IFN-β production was significantly increased in all three siRNA-treated cells compared to control siRNA-transfected cells (Fig. S2B), while poly(I:C)-induced IFN-β production was largely unaffected. Consistently, all three siRNAs increased dsDNA-induced IFN-β production at various time points after stimulation (Fig. S2C) but did not affect poly(I:C)-induced IFN-β production at any time point (Fig. S2D). Moreover, HSV-1- and VSV-induced IFN-β production was also markedly increased in all three siRNA-treated cells (Fig. S2E and F). All of these data indicate that suppression of PPP6C specifically enhances 5′ppp dsRNA- and dsDNA-induced but not poly(I:C)-induced innate immune responses. PPP6C siRNA 8 was used in subsequent experiments.

10.1128/mBio.01728-20.2 FIG S2 Knockdown of PPP6C enhances 5′ppp dsRNA- and dsDNA-induced but not poly(I:C)-induced IFN-β production. EA.hy926 cells were transfected with control NS or three individual PPP6C siRNAs (numbers 6, 7, and 8) for 72 h. (A) Cell lysates were immunoblotted with the indicated antibodies. (B) Cells were then transfected with poly(I:C) (0.5 μg/ml), 5′ppp dsRNA (4 μg/ml), or dsDNA (4 μg/ml) for 16 h. IFN-β production in the supernatant was measured by ELISA. (C to F) Cells were then transfected with dsDNA (C) or poly(I:C) (D) or infected with HSV-1 (MOI = 10) (E) or VSV (MOI = 10) (F) for the indicated time periods. IFN-β production in the supernatant was measured by ELISA. The data shown are representative of two independent experiments. *, *P* < 0.05 by Student’s *t* test. Download FIG S2, PDF file, 0.8 MB. Copyright © 2020 Ni et al. 2020 Ni et al. This content is distributed under the terms of the Creative Commons Attribution 4.0 International license.

### Deficiency of PPP6C enhances dsDNA-induced but not poly(I:C)-induced innate immune responses in primary HUVEC.

**FIG 2:**
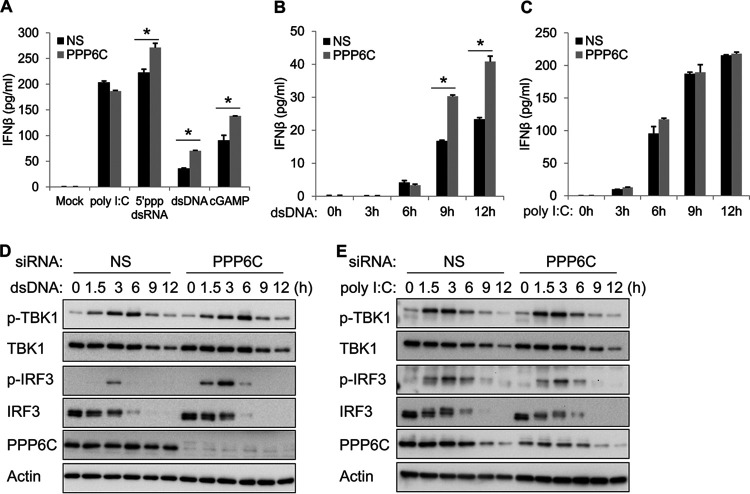
Deficiency of PPP6C enhances dsDNA- but not poly(I:C)-induced innate immune responses in primary HUVEC. (A) Primary HUVEC were transfected with NS or PPP6C siRNA for 72 h and then transfected with poly(I:C) (0.5 μg/ml), 5′ppp dsRNA (4 μg/ml), dsDNA (4 μg/ml), or cGAMP (4 μg/ml) for 16 h. IFN-β production in the supernatant was measured by ELISA. (B and C) Primary HUVEC were transfected with NS or PPP6C siRNA for 72 h and then transfected with dsDNA (B) or poly(I:C) (C) for the indicated time periods. IFN-β production in the supernatant was measured by ELISA. (D and E) Primary HUVEC were transfected with NS or PPP6C siRNA for 72 h and then transfected with dsDNA (D) or poly(I:C) (E) for the indicated time periods. Cell lysates were immunoblotted with the indicated antibodies. The data shown are representative of three independent experiments. Data in panels A to C are presented as mean + SD. *, *P* < 0.05 by Student's *t* test.

To evaluate the function of PPP6C in a relevant cell type, we transfected NS or PPP6C siRNA into primary human umbilical vein endothelial cells (HUVEC) for 72 h followed by stimulation with poly(I:C), 5′ppp dsRNA, dsDNA, or cGAMP. Knockdown of PPP6C in HUVEC significantly increased 5′ppp dsRNA-, dsDNA-, and cGAMP-induced IFN-β production but did not affect poly(I:C)-induced IFN-β production (Fig. 2A). dsDNA-induced IFN-β production was also enhanced at various time points after stimulation (Fig. 2B), while poly(I:C)-induced IFN-β production was not affected (Fig. 2C). Consistently, phosphorylation of IRF3 was markedly increased in PPP6C-depleted cells after dsDNA stimulation (Fig. 2D) but not in response to poly(I:C) stimulation (Fig. 2E). In summary, suppression of PPP6C expression in primary HUVEC showed a phenotype similar to that in EA.hy926 cells.

### PPP6C regulates dsDNA-induced IRF3 activation but not NF-κB activation.

**FIG 3:**
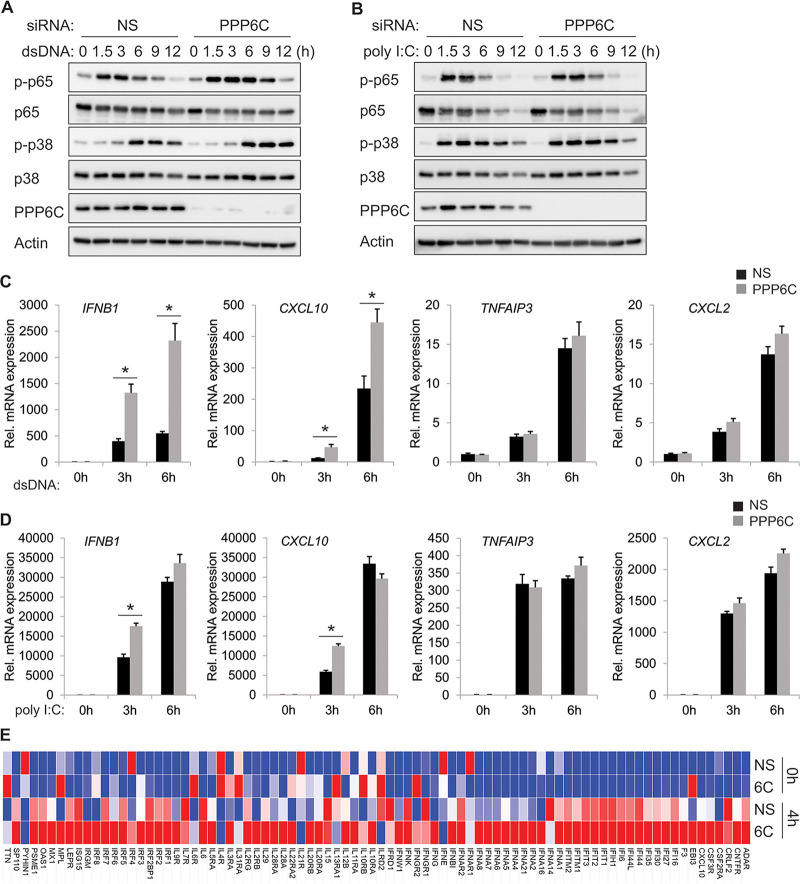
PPP6C regulates dsDNA-induced IRF3 activity but not NF-κB activity. (A and B) EA.hy926 cells were transfected with NS or PPP6C siRNA for 72 h and then transfected with dsDNA (A) or poly(I:C) (B) for the indicated times. Cell lysates were immunoblotted with the indicated antibodies. (C and D) EA.hy926 cells were transfected with NS or PPP6C siRNA for 72 h and then transfected with dsDNA (C) or poly(I:C) (D) for the indicated time periods. mRNA expression of the indicated genes was measured by real-time PCR, and fold changes were normalized to β-actin mRNA. (E) Heat map of the human interferon and receptor PCR array. EA.hy926 cells were transfected with NS or PPP6C siRNA for 72 h and then transfected with dsDNA for 0 or 4 h. RNA was extracted from the indicated samples, and mRNA levels of indicated genes were analyzed using the human interferon and receptor RT^2^ Profiler PCR array. mRNA levels of genes were normalized to β-actin. The heat map was generated using the web-based tool Morpheus (https://software.broadinstitute.org/morpheus/). The data shown are representative of three independent experiments, except in panel E, which is representative of two independent experiments. Data in panels C and D are presented as mean + SD. *, *P* < 0.05 by Student's *t* test. 6C, PPP6C.

Transcription of the IFN-β gene is regulated by the coordinated activation of a number of transcription factors, such as IRF3, IRF7, NF-κB, and activator protein-1 (AP-1) (26). Activation of either the RLR pathway or the cGAS-STING pathway is known to activate the TBK1-IRF3 and NF-κB axis (2). Since we have shown that PPP6C negatively regulates dsDNA-induced IRF3 activation, we further evaluated whether PPP6C also modulates STING-dependent activation of NF-κB and AP-1 signaling. Immunoblot analysis indicated that dsDNA-induced phosphorylation of p65 or p38 was not affected in PPP6C-silenced cells (Fig. 3A). Phosphorylation of p65 or p38 was not affected in these cells after stimulation with poly(I:C) either (Fig. 3B). Transcription of several type I IFN genes and proinflammatory cytokine genes, e.g., *IFNB1* and *CXCL10*, is regulated by IRF3, NF-κB, and AP-1 together, while others are regulated only by NF-κB and AP-1 but not IRF3, e.g., *TNFAIP3* and *CXCL2* (11, 17). To further confirm that PPP6C regulates only dsDNA-induced IRF3 activation but not NF-κB activation, we measured the mRNA expression levels of *IFNB1*, *CXCL10*, *TNFAIP3*, and *CXCL2* in PPP6C siRNA-treated EA.hy926 cells following dsDNA stimulation. Induction of both *IFNB1* and *CXCL10* mRNA was significantly increased in PPP6C-deficient cells, while expression of *TNFAIP3* and *CXCL2* mRNA was the same in control cells and PPP6C-deficient cells (Fig. 3C). Depletion of PPP6C did not affect the expression of any of the four genes after poly(I:C) stimulation for 6 h (Fig. 3D). However, mRNA expression of *IFNB1* and *CXCL10* genes did increase significantly at 3 h after poly(I:C) stimulation (Fig. 3D). In contrast, phosphorylation of p65 was increased in PPP6C knockdown cells in response to 5′ppp dsRNA stimulation (Fig. S3A). Enhanced NF-κB activation led to increased induction of *TNFAIP3* and *CXCL2* genes, although only at 6 h after 5′ppp dsRNA stimulation (Fig. S3B). To extend these studies, we used a PCR array for human interferons and receptors (Qiagen) to analyze 84 genes whose expression is controlled by, or involved in, cell signaling mediated by IFN ligands and receptors. EA.hy926 cells were transfected with NS or PPP6C siRNA for 72 h, followed by dsDNA stimulation for 4 h. mRNA expression of 30 genes was increased by more than 2-fold in NS siRNA-treated cells (4 h versus 0 h). Among these genes, 27 of them showed a higher induction in PPP6C siRNA-transfected cells than in control siRNA-transfected cells (4 h, PPP6C versus NS). The overall gene expression profile of all 84 genes tested is shown as a heat map in Fig. 3E, and the raw data are available in Table S1.

10.1128/mBio.01728-20.3 FIG S3 Suppression of PPP6C moderately increases 5′ppp dsRNA-induced NF-κB activity. (A) EA.hy926 cells were transfected with NS or PPP6C siRNA for 72 h and then transfected with 5′ppp dsRNA for the indicated time points. Cell lysates were immunoblotted with the indicated antibodies. (B) EA.hy926 cells were transfected with NS or PPP6C siRNA for 72 h and then transfected with 5′ppp dsRNA for the indicated times. mRNA expression of the indicated genes was measured by real-time PCR, and fold changes were normalized to β-actin mRNA. The data shown are representative of two independent experiments. Data in panel B are means and SD. *, *P* < 0.05 by Student’s *t* test. Download FIG S3, PDF file, 0.7 MB. Copyright © 2020 Ni et al. 2020 Ni et al. This content is distributed under the terms of the Creative Commons Attribution 4.0 International license.

10.1128/mBio.01728-20.5 TABLE S1 PCR array for human interferons and receptors in EA.hy926 cells. The raw data were used to generate the heat map in Fig. 3E. Download Table S1, PDF file, 0.1 MB. Copyright © 2020 Ni et al. 2020 Ni et al. This content is distributed under the terms of the Creative Commons Attribution 4.0 International license.

### Knockdown of PPP6C enhances HSV-1- and VSV-induced innate immune responses, which impairs virus replication.

**FIG 4:**
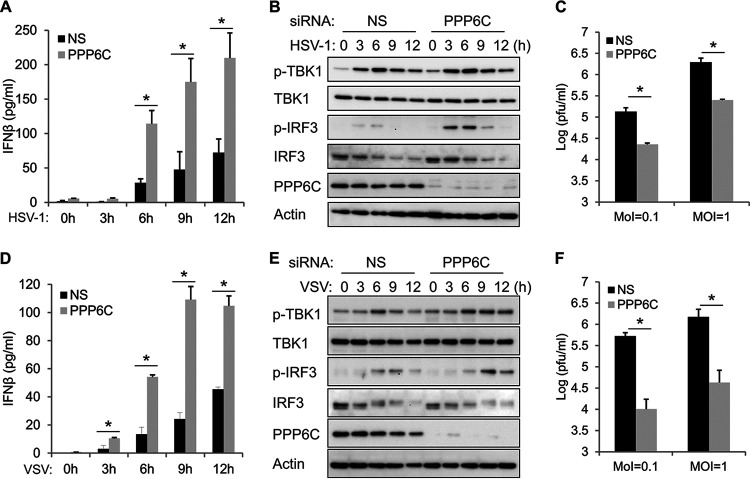
Knockdown of PPP6C enhances HSV-1- and VSV-induced innate immune responses and inhibits virus replication. (A and B) EA.hy926 cells were transfected with NS or PPP6C siRNA for 72 h and then infected with HSV-1 (MOI = 10) for the indicated times. IFN-β production in the supernatant was measured by ELISA (A), and cell lysates were immunoblotted with the indicated antibodies (B). (C) EA.hy926 cells were transfected with NS or PPP6C siRNA for 72 h and then infected with HSV-1 (MOI = 0.1 or 1) for 24 h. The viral titer in the supernatant was measured by plaque assay. (D and E) EA.hy926 cells were transfected with NS or PPP6C siRNA for 72 h and then infected with VSV (MOI = 10) for the indicated time periods. IFN-β production in the supernatant was measured by ELISA (D), and cell lysates were immunoblotted with the indicated antibodies (E). (F) EA.hy926 cells were transfected with NS or PPP6C siRNA for 72 h and then infected with VSV (MOI = 0.1 or 1) for 24 h. The viral titer in the supernatant was measured by plaque assay. The data shown are representative of three independent experiments. Data in panels A, C, D, and F are presented as mean + SD. *, *P* < 0.05 by Student's *t* test.

To further investigate the role of PPP6C in regulating innate immune responses against pathogen invasion, we infected PPP6C knockdown or control EA.hy926 cells with a DNA virus, HSV-1. ELISA analysis indicated that PPP6C deficiency enhances HSV-1-induced IFN-β production (Fig. 4A). Consistently, slightly increased TBK1 phosphorylation and markedly increased IRF3 phosphorylation were seen in PPP6C-deficient cells in response to HSV-1 infection (Fig. 4B). Because of the enhanced innate immune response, HSV-1 replication was significantly inhibited in PPP6C-suppressed cells compared to control cells (Fig. 4C). We also evaluated the role of PPP6C during RNA virus infection and found that knockdown of PPP6C similarly enhanced VSV-induced IFN-β production (Fig. 4D) and TBK1-IRF3 activation (Fig. 4E), which led to impaired virus replication in PPP6C-deficient cells compared to control cells (Fig. 4F).

### Suppression of PPP6C expression inhibits KSHV lytic reactivation due to the increased innate immune response.

**FIG 5:**
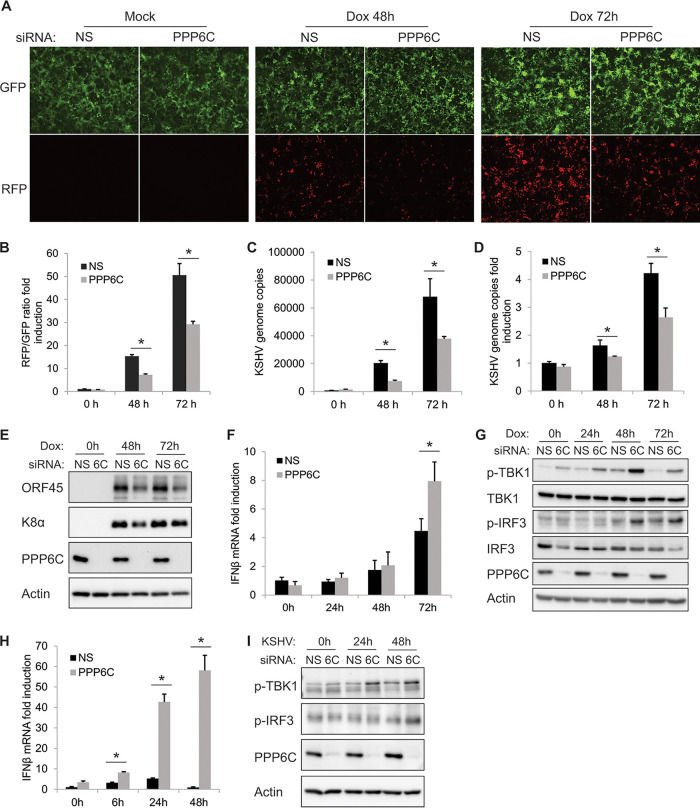
Suppression of PPP6C inhibits KSHV lytic reactivation due to an increased innate immune response. iSLK.219 cells were transfected with NS siRNA or PPP6C siRNA for 48 h and then treated with doxycycline (Dox; 1 μg/ml). (A) GFP- and RFP-positive cells were imaged at 0, 48, and 72 h after Dox treatment, and a representative image of each sample is shown. (B) GFP and RFP fluorescence intensities were measured by a Clariostar plate reader, and the RFP/GFP ratio was calculated at the indicated time points. (C) KSHV genome copy numbers in the supernatants were measured by real-time PCR. (D) Relative KSHV genome copy numbers in the cells were measured by real-time PCR and normalized to β-actin. (E) Cell lysates were collected at the indicated time points, and immunoblots were performed with the indicated antibodies. (F) IFN-β mRNA fold induction was measured by real-time PCR and normalized to β-actin mRNA. (G) Cell lysates were collected at the indicated time points, and immunoblots were performed with the indicated antibodies. (H and I) EA.hy926 cells were transfected with NS or PPP6C siRNA for 72 h and then infected with KSHV for the indicated times. IFN-β mRNA fold induction was measured by real-time PCR (H), and cell lysates were immunoblotted with the indicated antibodies (I). The data shown are representative of three independent experiments. Data in panels B, C, D, F, and H are presented as mean + SD. *, *P* < 0.05 by Student's *t* test. 6C, PPP6C.

We next investigated how PPP6C is involved in modulating KSHV reactivation. We knocked down PPP6C in iSLK.219 cells, which contain a latent KSHV genome and a doxycycline (Dox)-inducible RTA (replication and transcription activator) protein to enable entry into the lytic cycle. The cells also express a constitutive green fluorescent protein (GFP) marker and a red fluorescent protein (RFP) marker driven by a lytic cycle-specific promoter, which can be used as an indicator for reactivated lytic cells (27). We found that RFP-positive cells were markedly reduced after Dox treatment in PPP6C-deficient cells compared to control cells (Fig. 5A), indicating that suppression of PPP6C inhibited KSHV lytic reactivation. The RFP and GFP fluorescence intensities in the cells were further quantified by a plate reader and are presented as fold induction of the RFP/GFP ratio (Fig. 5B). Consistent with these data, we found that suppression of PPP6C significantly reduced numbers of KSHV genome copies in the supernatant (Fig. 5C) as well as in the cells (Fig. 5D). We also checked the expression of KSHV viral proteins ORF45 and K8α and found that expression of both proteins was reduced in PPP6C-deficient cells (Fig. 5E). We further demonstrated that the inhibition of KSHV reactivation in PPP6C-silenced cells was likely due to enhanced innate immune responses, because IFN-β mRNA induction (Fig. 5F) as well as phosphorylation of TBK1 and IRF3 (Fig. 5G) was increased in the absence of PPP6C. Moreover, PPP6C depletion also enhanced KSHV primary infection-induced IFN-β mRNA induction (Fig. 5H) and phosphorylation of TBK1 and IRF3 (Fig. 5I) in EA.hy926 cells compared to those in control cells. In general, PPP6C deficiency leads to an enhanced induction of type I IFN signaling and thereby inhibits KSHV lytic reactivation.

### PPP6C negatively regulates the cGAS-STING signaling pathway by dephosphorylating STING.

**FIG 6:**
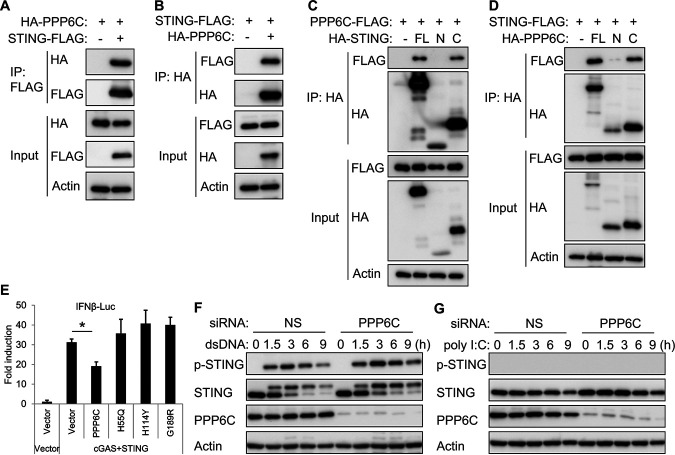
PPP6C interacts with STING and dephosphorylates STING. (A and B) HA-tagged STING and FLAG-tagged PPP6C were transfected individually or together into HEK293T cells for 30 h. Cell lysates were immunoprecipitated with anti-FLAG (A) or anti-HA (B) antibodies and then immunoblotted with the indicated antibodies. (C) FLAG-tagged PPP6C was cotransfected with full-length (FL), N-terminal (N; aa 1 to 195), or C-terminal (C; aa 181 to 379) HA-tagged STING into HEK293T cells for 30 h. Cell lysates were immunoprecipitated with anti-HA antibody and then immunoblotted with the indicated antibodies. (D) FLAG-tagged STING was cotransfected with full-length (FL), N-terminal (N; aa 1 to 160), or C-terminal (C; aa 151 to 305) HA-tagged PPP6C into HEK293T cells for 30 h. Cell lysates were immunoprecipitated with anti-HA antibody and then immunoblotted with the indicated antibodies. (E) HEK293T cells were cotransfected as indicated with IFN-β-luc, pRL-TK, cGAS and STING plasmids along with plasmids expressing wild-type PPP6C or the indicated PPP6C mutants. Luciferase activity was measured after 30 h. (F and G) EA.hy926 cells were transfected with NS or PPP6C siRNA for 72 h and then transfected with dsDNA (F) or poly(I:C) (G) for the indicated times. Cell lysates were immunoblotted with the indicated antibodies. The data shown are representative of three independent experiments. Data in panel E are presented as mean + SD. *, *P* < 0.05 by Student's *t* test.

We next investigated the mechanism of how PPP6C regulates the cGAS-STING pathway. Considering that PPP6C is a phosphatase, we hypothesized that PPP6C may dephosphorylate certain components of this pathway. To identify the target of PPP6C, we overexpressed the hemagglutinin (HA)-tagged PPP6C along with FLAG-tagged RIG-I, MAVS, cGAS, STING, TBK1 or IRF3 in HEK293T cells and then performed an immunoprecipitation using anti-FLAG beads. Immunoblot analysis showed that PPP6C interacts with RIG-I, MAVS, and STING, but not the other proteins (Fig. S4A). We also performed a luciferase assay which indicated that PPP6C inhibits RIG-I-, MAVS-, and STING-induced IFN-β promoter-driven luciferase activity but not that induced by TBK1 or IRF3 (Fig. S4B). These data suggest that PPP6C probably acts on STING in the cGAS-STING pathway. We further confirmed the interaction between PPP6C and STING by immunoprecipitation assays (Fig. 6A and B). By using HA-tagged STING truncation variants, we determined that the C-terminal region of STING was associated with PPP6C (Fig. 6C). Likewise, the C-terminal region of PPP6C was mapped as facilitating the interaction of PPP6C with STING (Fig. 6D). A previous study reported that some point mutations in the PPP6C protein could impair its phosphatase activity (22). We constructed three catalytically compromised PPP6C mutants and found that wild-type PPP6C, but not these PPP6C mutants, reduced cGAS-STING-induced IFN-β promoter-driven luciferase activity (Fig. 6E), indicating that the phosphatase activity is required for PPP6C to regulate STING function. Furthermore, knockdown of PPP6C greatly increased STING phosphorylation at Ser366 after dsDNA stimulation (Fig. 6F), but not after poly(I:C) stimulation (Fig. 6G), which does not activate the STING-dependent pathway. These data suggest that PPP6C negatively regulates the activation of the cGAS-STING pathway by interacting with, and dephosphorylating, STING.

10.1128/mBio.01728-20.4 FIG S4 PPP6C interacts with STING. (A) HEK293T cells were cotransfected with HA-tagged PPP6C and plasmids expressing the indicated FLAG-tagged proteins for 30 h. Cell lysates were immunoprecipitated with anti-FLAG antibody and then immunoblotted with the indicated antibodies. (B) HEK293T cells were cotransfected as indicated with IFN-β-luc, pRL-TK and plasmids expressing the indicated proteins. Luciferase activity was measured after 30 h. ΔRIG-I is a constitutively active form of RIG-I (aa 1 to 229). IRF3sa refers to IRF3 S396D, a superactive form of IRF3. The data shown are representative of three independent experiments. Data in panel B are means and SD. *, *P* < 0.05 by Student’s *t* test. Download FIG S4, PDF file, 0.6 MB. Copyright © 2020 Ni et al. 2020 Ni et al. This content is distributed under the terms of the Creative Commons Attribution 4.0 International license.

## DISCUSSION

The cGAS-STING pathway plays a crucial role in activating the host defense against invasion of DNA viruses and other pathogens. Many viruses have evolved different mechanisms to antagonize the cGAS-STING pathway in order to facilitate their replication (28). A previous study in our lab identified KSHV ORF48 as a negative regulator of the cGAS-STING pathway in a luciferase reporter assay-based screen, although the exact mechanism was unclear (25). In this study, we identified PPP6C and four regulatory subunits as ORF48-interacting proteins by mass spectrometry analysis. We further demonstrated that PPP6C negatively regulates the cGAS-STING pathway, as PPP6C deficiency greatly enhanced dsDNA-induced IRF3 activation and type I IFN production. During the revision of our manuscript, another group reported that PPP6C inhibits DNA-induced innate immune responses (29).

It is possible that ORF48 antagonizes the cGAS-STING pathway by recruiting PPP6C, although it may also function in a PPP6C-independent manner. The detailed mechanism will be investigated in the future. Activation of the cGAS-STING pathway by cytosolic DNA quickly leads to induction of hundreds of type I IFN genes and proinflammatory cytokine genes within hours (11). Our PCR array data demonstrate that in addition to IFN-β, PPP6C deficiency also broadly increases the induction of many other IFN and cytokine genes, such as genes for CXCL10, IFN-α family members, and several interleukins, in response to DNA stimulation (Fig. 3E and Table S1).

Depletion of PPP6C also enhanced IRF3 activation and IFN-β production in response to 5′ppp dsRNA stimulation. However, poly(I:C)-induced IFN-β production was largely not affected in the absence of PPP6C, although a temporary increase of *IFNB1* and *CXCL10* mRNA expression was seen in the early stages following stimulation. 5′ppp dsRNA is the ligand for RIG-I, while poly(I:C) (high molecular weight) is sensed mainly by MDA5. Thus, our data suggest that PPP6C differentially regulates RIG-I-dependent and MDA5-dependent RNA sensing, although both receptors cooperate with MAVS to activate downstream signaling. Further investigation is needed to unveil the detailed mechanism. A previous study reported that PPP6C positively regulates the RNA sensing pathway by dephosphorylating RIG-I and that PPP6C deficiency inhibits VSV-induced IFN-β production (30). However, our data show that PPP6C inhibits MAVS-induced IFN-β–luciferase activity, suggesting that PPP6C may act downstream of RIG-I. Our study also shows that PPP6C negatively regulates not only the innate immune responses induced by the DNA virus, HSV-1, but also those induced by the RNA virus, VSV. Thus, PPP6C plays a proviral role during virus infection. Additionally, we demonstrated that PPP6C also facilitates KSHV lytic reactivation from latency. Moreover, our data are supported by another study showing that PPP6C promotes the replication of influenza A virus (31), which is known to be sensed by RIG-I.

PPP6C has been previously reported to suppress NF-κB signaling by either stabilizing IκBε or by suppressing sustained TAK1 activation (23, 24). In the context of DNA sensing and viral infection, our data show that although PPP6C deficiency enhanced dsDNA-induced IRF3 activation, PPP6C did not seem to affect p65 phosphorylation or NF-κB-regulated gene expression induced by cytosolic dsDNA, suggesting that PPP6C is not involved in regulating STING-dependent activation of NF-κB. A previous study showed that phosphorylation of STING at Ser366 affects only STING-dependent IRF3 activation, not NF-κB activation (11). Therefore, our data further support this finding, as dephosphorylation of STING by PPP6C also differentially regulates STING-dependent IRF3 activation and NF-κB activation.

Mass spectrometry analysis revealed that human STING is phosphorylated at several serine residues in the C terminus (11). Aside from Ser366, the function of phosphorylation at the other serine residues is still unknown. The function of phosphorylation at STING Ser366 still needs to be elucidated. One group reported that TBK1 phosphorylates STING at Ser366, which is required for further activation of IRF3 (16, 32). While another study suggests that phosphorylation at STING Ser366 is mediated by ULK1 and that this phosphorylation prevents sustained STING activity (11). Our data show that loss of PPP6C increases dsDNA-induced STING phosphorylation at Ser366, which subsequently enhances IRF3 activation and induction of innate immune genes. Therefore, our findings support the model that phosphorylation at STING Ser366 is required for activation of the STING-dependent DNA sensing pathway.

In summary, our data indicate that PPP6C negatively regulates the cGAS-STING pathway by removing STING phosphorylation at Ser366. Therefore, PPP6C helps prevent the sustained production of STING-dependent cytokines, which would otherwise lead to severe autoimmune disease. Our study reveals additional mechanisms for the regulation of cytosolic DNA sensing pathway and might assist the development of novel therapeutics designed to prevent a variety of self-DNA triggered disorders.

## MATERIALS AND METHODS

### Cells and viruses.

HEK293T cells, Vero cells, BHK cells and HUVEC were purchased from the American Type Culture Collection (ATCC). EA.hy926 cells were purchased from the tissue culture facility at the University of North Carolina at Chapel Hill. 293FT cells were purchased from Thermo Fisher. iSLK.219 cells were a kind gift from Don Ganem. HEK293T cells, Vero cells, BHK cells, 293FT cells, and EA.hy926 cells were maintained in Dulbecco’s minimal essential medium (DMEM) (Corning) containing 10% fetal bovine serum (FBS) (Sigma) and 1% penicillin-streptomycin (Pen-Strep) (Corning). HUVEC were maintained in EGM-2 supplied with growth factors obtained from the EGM-2 Bullet kit (Lonza). iSLK.219 cells were maintained in DMEM containing 10% tetracycline (Tet)-free FBS (Sigma), 1% Pen-Strep, 10 μg/ml puromycin (Corning), 50 μg/ml Geneticin (Corning), and 100 μg/ml hygromycin B (Corning). All cells were maintained at 37°C in a 5% CO_2_ laboratory incubator which was subject to routine cleaning and decontamination. HSV-1 (KOS strain) was obtained from ATCC and propagated in Vero cells. VSV was a kind gift from Doug Lyles and was propagated in BHK cells. KSHV was purified form iSLK.219 cells in our lab.

### Reagents and antibodies.

Interferon-stimulatory DNA (90-mer) used as dsDNA in this study was prepared as previously described (17). Poly(I:C) (high molecular weight), 5′ppp dsRNA, and 2′3′-cGAMP were purchased from InvivoGen. Doxycycline hydrochloride was purchased from Fisher Scientific. Anti-FLAG M2 affinity gel was purchased from Sigma-Aldrich. Antibodies used in the study and their sources were as follows: anti-PPP6C (A300-844A; Bethyl Laboratories), anti-IRF3 (4302; Cell Signaling), anti-phospho-IRF3 (29047; Cell Signaling), anti-TBK1 (3504; Cell Signaling), anti-phospho-TBK1 (5483; Cell Signaling), anti-p65 (8242; Cell Signaling), anti-phospho-p65 (3033; Cell Signaling), anti-p38 (9212; Cell Signaling), anti-phospho-p38 (4511; Cell Signaling), anti-STING (13647; Cell Signaling), anti-phospho-STING (50907; Cell Signaling), anti-HA conjugated to horseradish peroxidase (HRP) (14031; Cell Signaling), anti-HA (H9658; Sigma), anti-FLAG (F1804; Sigma), anti-FLAG–HRP (A8592; Sigma), anti-actin–HRP (sc-47778 HRP; Santa Cruz), anti-KSHV ORF45 (MA5-14769; Thermo Fisher), and anti-KSHV K8alpha (sc-57889; Santa Cruz).

### Plasmid construction.

pcDNA4/TO-ORF48-Strep was a kind gift from Britt A. Glaunsinger at the University of California, Berkeley. HA-PPP6C was a kind gift from David L. Brautigan at the University of Virginia. PPP6C-FLAG and HA-PPP6C truncated mutants were cloned into pcDNA3.1 (Invitrogen) using a standard molecular cloning protocol. PPP6C H55Q, H114Y, and G189R mutants were generated using a QuikChange Lightning site-directed mutagenesis kit (Agilent). HA-STING, HA-STING-N (amino acids [aa] 1 to 195) and HA-STING-C (aa 181 to 379) were kind gifts from Glen Barber at the University of Miami. pRL-TK plasmid was obtained from Promega. IFN-β-luc plasmid was a generous gift from Zhijian Chen at the University of Texas Southwestern Medical Center. pCIG2-PURO-FLAG, ΔRIG-I, MAVS, and TBK1 plasmids were kind gifts from Jenny Ting at the University of North Carolina at Chapel Hill. pUNO1-cGAS and pUNO-IRF3sa were purchased from InvivoGen.

### Lentivirus infections and stable cell line generation.

ORF48 was cloned into lentivirus-based vector pCIG2-PURO-FLAG using a standard molecular cloning protocol, and lentiviral particles were produced in 293FT cells using a Virapower lentiviral expression system (Invitrogen) according to the manufacturer’s protocol. For lentiviral transductions, EA.hy926 cells were grown to 70% confluence and inoculated with lentivirus in the presence of 8 μg/ml Polybrene. Medium containing virus was removed after 24 h, and cells were cultured in fresh medium for another 24 h. Appropriate medium with 1 μg/ml puromycin was added 48 h after lentivirus transduction for selection. The medium was changed every 3 days, and stable cell lines were maintained in medium with 1 μg/ml puromycin to retain protein expression.

### Mass spectrometry analysis.

Four 150-mm dishes of EA.hy926 ORF48-FLAG cells or control cells were cultured until 90% confluence and lysed using 0.1% Nonidet P-40 (NP-40) buffer (50 mM Tris-HCl [pH 7.6], 150 mM NaCl, 0.1% NP-40) with protease inhibitor cocktails (Sigma). Immunoprecipitation was performed using anti-FLAG M2 affinity gel, and the beads were washed four times with lysis buffer before on-bead digestion. The detailed mass spectrometry analysis and data filter were described previously (33). Briefly, reverse-phase nano-high-performance liquid chromatography (nano-HPLC) coupled with a nanoACQUITY ultraperformance liquid chromatography (UPLC) system (Waters Corporation, Milford, MA) was used to separate trypsinized peptides. Peptide analysis was performed on an Orbitrap Elite mass spectrometer (Thermo Scientific). The UPLC method, MS data acquisition, and ion source settings were published previously (34). All raw mass spectrometry data were searched using MaxQuant version 1.5.2.6.

### DNA transfection and RNA interference.

Plasmids, poly(I:C), 5′ppp dsRNA, dsDNA, and cGAMP transfection into HEK293T cells, EA.hy926 cells, and primary HUVEC were carried out using Lipofectamine 2000 (Invitrogen) with Opti-MEM (Invitrogen) according to the manufacturer’s instructions. siRNA transfections were performed using Lipofectamine RNAiMAX (Invitrogen) with Opti-MEM. The nontargeting control (NS) siRNA and PPP6C siRNA were purchased from Dharmacon, and the siRNA sequences are as follows: NS, UGGUUUACAUGUCGACUAA; PPP6C 6, CUAAAUGGCCUGAUCGUAU; PPP6C 7, CGCUAGACCUGGACAAGUA; and PPP6C 8, GUUUGGAGACCUUCACUUA.

### Immunoblotting and immunoprecipitation.

Cell lysates boiled in sodium dodecyl sulfate (SDS) loading buffer were separated by standard SDS-polyacrylamide gel electrophoresis (PAGE), transferred to polyvinylidene difluoride (PVDF) membranes (Millipore), and immunoblotted with primary antibodies. For immunoprecipitation, whole-cell lysate were generated by lysing cells in ice-cold NP-40 buffer (50 mM Tris-HCl [pH 7.6], 150 mM NaCl, 1% NP-40, 1 mM EDTA) with protease inhibitor cocktails (Sigma) at 4°C followed by centrifugation. The lysates were incubated with the indicated antibodies overnight at 4°C, and then 30 μl protein A/G beads was added for another 2 h. The beads were washed four times with lysis buffer and boiled in an appropriate amount of 2× sample buffer before being analyzed by SDS-PAGE.

### Quantitative real-time PCR and PCR arrays.

Total RNA was extracted with an RNeasy Plus minikit (Qiagen), and cDNA synthesis was performed using a SensiFAST cDNA synthesis kit (Bioline). Real-time qPCR was performed with Sensi-Fast SYBR (Thomas Scientific). The housekeeping gene *ACTB* was used for normalization. Primers used in the study are as follows: human *IFNB1*, 5′-CTAACTGCAACCTTTCGAAGC-3′ and 5′-GGAAAGAGCTGTAGTGGAGAAG-3′; human *CXCL10*, 5′-CCTTATCTTTCTGACTCTAAGTGGC-3′ and 5′-ACGTGGACAAAATTGGCTTG-3′; human *TNFAIP3*, 5′-GATAGAAATCCCCGTCCAAGG-3′ and 5′-CTGCCATTTCTTGTACTCATGC-3′; human *CXCL2*, 5′-AACCGAAGTCATAGCCACAC-3′ and 5′-CTTCTGGTCAGTTGGATTTGC-3′; human *ACTB*, 5′-AAGACCTGTACGCCAACACA-3′ and 5′-AGTACTTGCGCTCAGGAGGA-3′.

Total RNA was extracted from EA.hy926 cells, and the subsequent cDNA synthesis was performed as described above. An RT^2^ Profiler PCR array for human interferons and receptors was purchased from Qiagen (catalog number PAHS-064Z); this array contains 84 primer sets against human interferon signaling-related genes and 5 housekeeping gene primer sets. Real-time PCR was then performed according to the manufacturer’s protocol. Fold change was calculated as the normalized gene expression (2^−ΔΔ^*^CT^*) in the test sample divided by the normalized gene expression (2^−ΔΔ^*^CT^*) in the control sample. The heat map was generated using the web-based tool Morpheus (https://software.broadinstitute.org/morpheus/).

### ELISA.

IFN-β concentrations in the supernatants from EA.hy926 cells or primary HUVEC were measured using the human IFN-β DuoSet ELISA kit (R&D Systems) according to the manufacturer’s instructions. Values were calculated from a standard curve derived from recombinant IFN-β provided in the kit and are presented as means and standard deviations (SD), in picograms per milliliter.

### Virus infection and plaque assay.

EA.hy926 cells were infected with HSV-1 or VSV at a multiplicity of infection (MOI) of 0.1 or 1 for 24 h. Supernatants from infected cells were harvested for plaque assay. Briefly, the supernatants were diluted with DMEM at a 10-fold serial dilution and then used to infect Vero cells (for HSV-1) or BHK cells (for VSV) for 1 h. The cells were then layered with complete DMEM containing 1% low-melting-point agarose gel and incubated at 37°C for another 2 days (for HSV-1) or 1 day (for VSV). Agarose gels were removed by heating in a microwave, and cells were fixed with a solution containing 0.1% crystal violet and 30% methanol. Plaques in appropriate wells were counted, and viral titers were calculated and are presented as PFU per milliliter.

### KSHV reactivation and fluorescence microscopy.

iSLK.219 cells were transfected with NS or PPP6C siRNA using Lipofectamine RNAiMax. At 48 h posttransfection, cells were replenished with complete DMEM containing 1 μg/ml doxycycline for KSHV reactivation. GFP and RFP images of the cells were taken with a fluorescence microscope at 48 and 72 hours after doxycyline treatment using the same exposure time setting. One representative image of each sample is shown. The RFP and GFP fluorescence intensity in each well (triple wells for each sample) was then measured by a microplate reader right after the microscopy. The fold induction was calculated and normalized to NS-treated cells without doxycycline treatment.

### Dual-luciferase-reporter assays.

HEK293T cells were cotransfected with 100 ng of IFN-β-luc reporter plasmid, 50 ng of pRL-TK reporter plasmid (HSV thymidine kinase promoter-driven renilla luciferase; internal control), and control empty vector or various expression plasmids. Thirty hours after transfection, luciferase activity was measured using a dual-luciferase-reporter assay system (Promega) according to the manufacturer’s instructions.

### Quantification and statistical analysis.

For all figures, values are means, and error bars show SD. Statistical significance of differences in mRNA expression level, IFN-β concentration, viral titers, and numbers of viral genome copies was determined using Student's *t* test (two-tailed). For all tests, a *P* value of <0.05 (indicated by asterisks in the figures) was considered statistically significant.
