# Supplementary material for: PPP6C Negatively Regulates STING-Dependent Innate Immune Responses
Source: mBio. 2020 Aug 4;11(4):e01728-20. doi: 10.1128/mBio.01728-20 (PMC7407089; doi:10.1128/mBio.01728-20)

A

Gene name	Protein name	Protein ID	LFQ intensity EV_mock	LFQ intensity ORF48_mock	ORF48 mock LFQ - EV mock LFQ
HHV-8 ORF48	E5LBX0_HHV8	E5LBX0	20.87479	31.26293	10.38814
ANKRD28	Serine/threonine-protein phosphatase 6 regulatory ankyrin repeat subunit A	Q8N8A2	20.23853	27.81684	7.57831
PPP6R3	Serine/threonine-protein phosphatase 6 regulatory subunit 3	Q5H9R7	21.17957	27.98961	6.81004
PPP6R1	Serine/threonine-protein phosphatase 6 regulatory subunit 1	Q9UPN7	21.02491	27.60003	6.57512
RB1CC1	RB1-inducible coiled-coil protein 1	Q8TDY2	20.67739	27.24191	6.56452
MAPK7	Mitogen-activated protein kinase 7	Q13164	19.85363	26.16212	6.30849
PPP6R2	Serine/threonine-protein phosphatase 6 regulatory subunit 2	O75170	19.56833	25.18733	5.619
SNRPD1	Small nuclear ribonucleoprotein Sm D1	P62314	21.62414	27.23526	5.61112
PPP6C	Serine/threonine-protein phosphatase 6 catalytic subunit	O00743	20.00804	25.56165	5.55361
PRKDC	DNA-dependent protein kinase catalytic subunit	P78527	23.56326	28.26217	4.69891

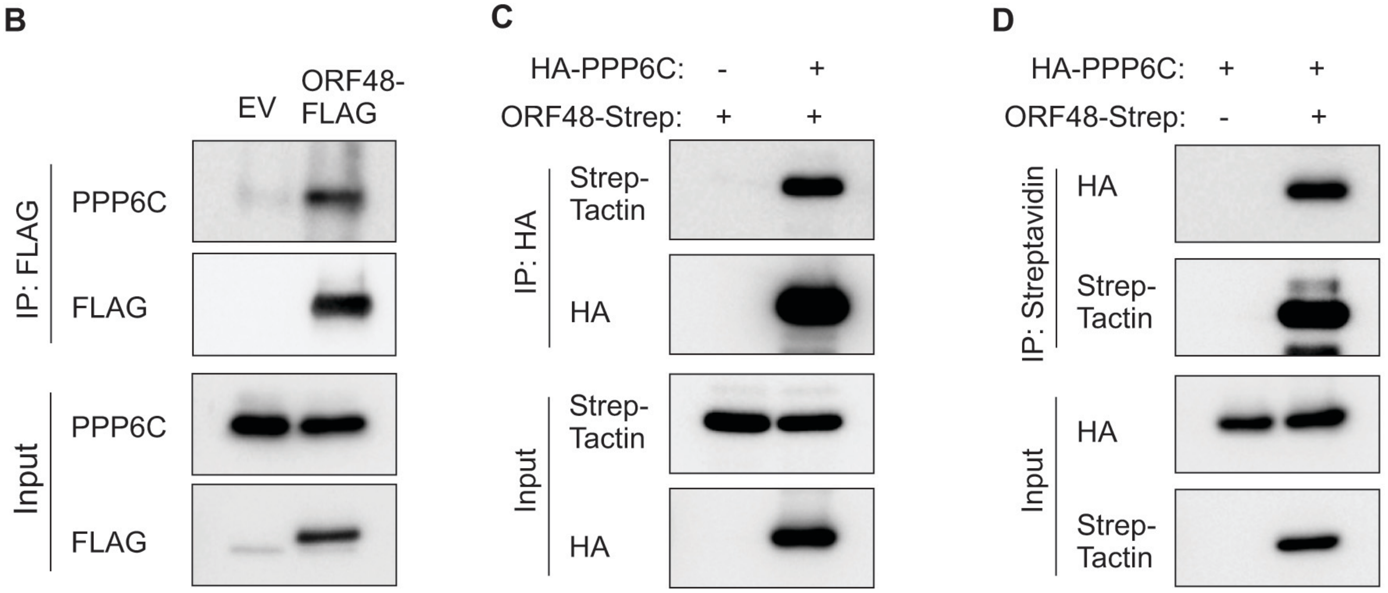

Supplement: FIG S1 [file mBio.01728-20-sf001.pdf]
