# Supplementary figures and images for: PPP6C Negatively Regulates STING-Dependent Innate Immune Responses
Source: mBio. 2020 Aug 4;11(4):e01728-20. doi: 10.1128/mBio.01728-20 (PMC7407089; doi:10.1128/mBio.01728-20)

**A**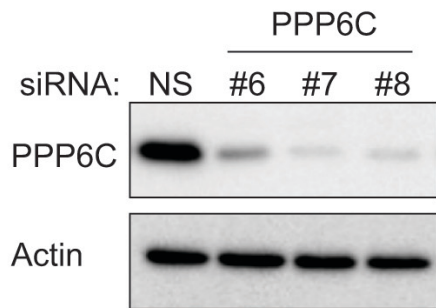**B**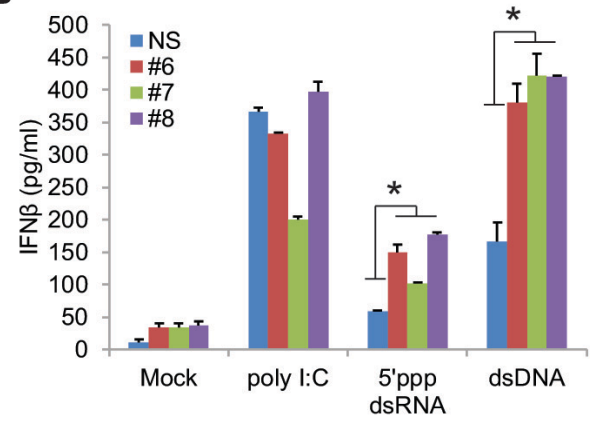**C**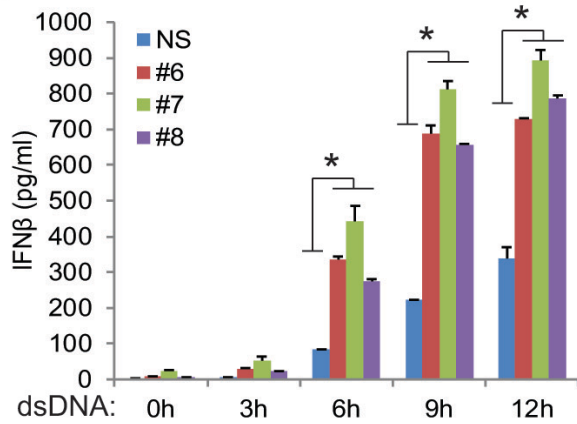**D**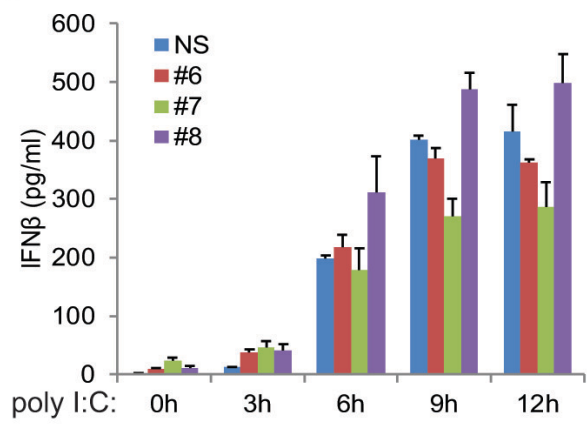**E**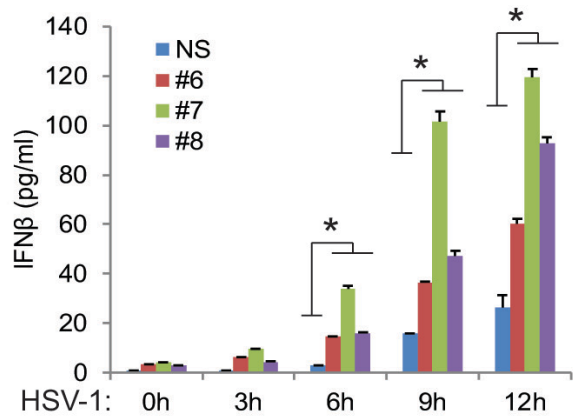**F**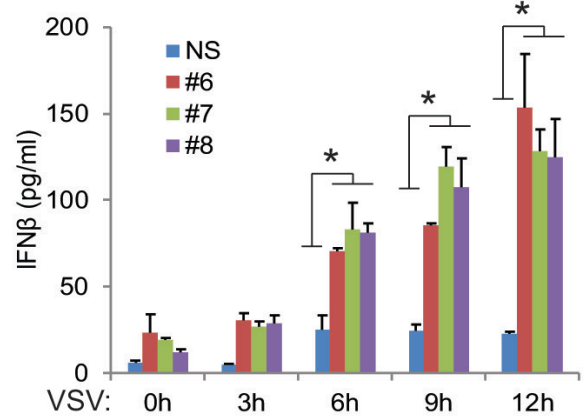

Supplement: FIG S2 [file mBio.01728-20-sf002.pdf]

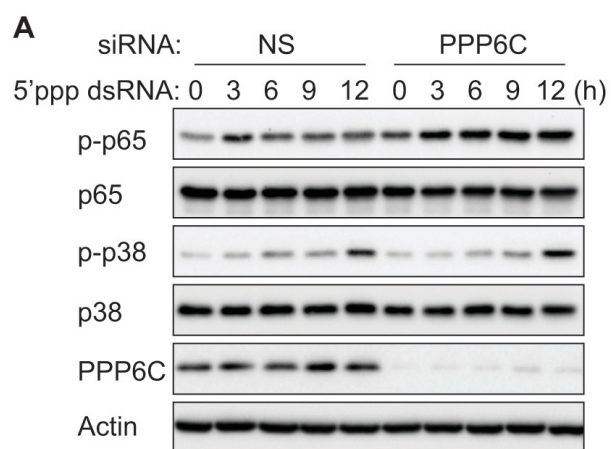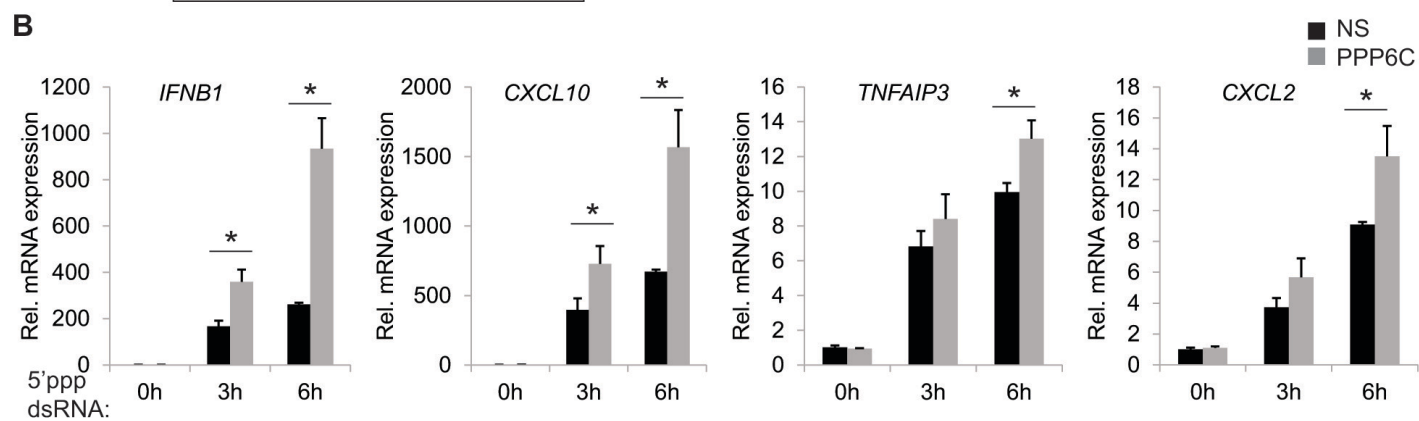

Supplement: FIG S3 [file mBio.01728-20-sf003.pdf]

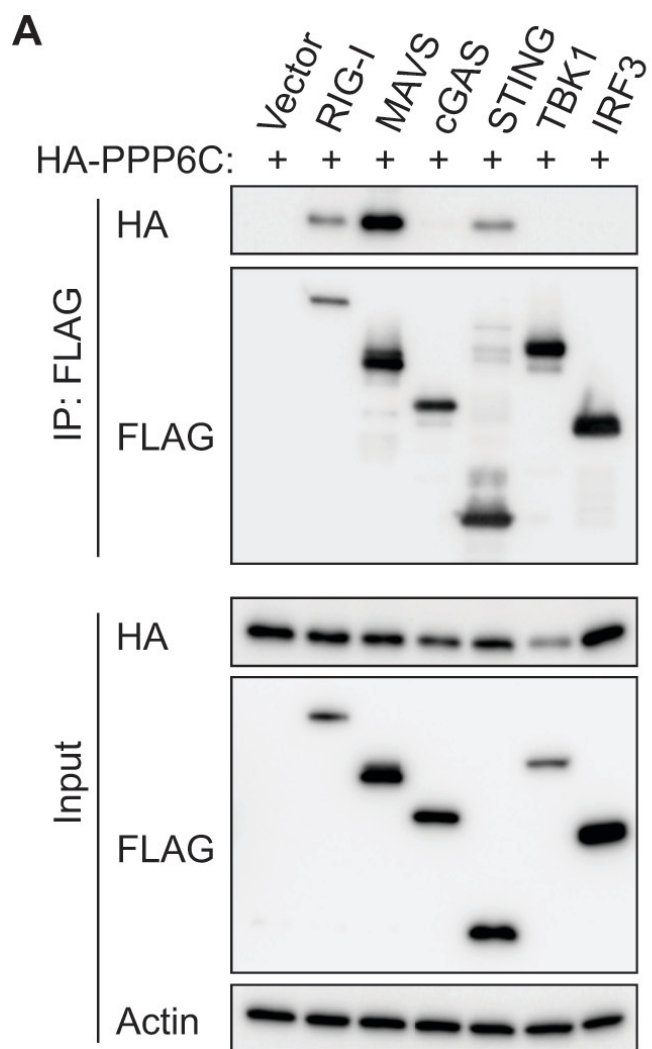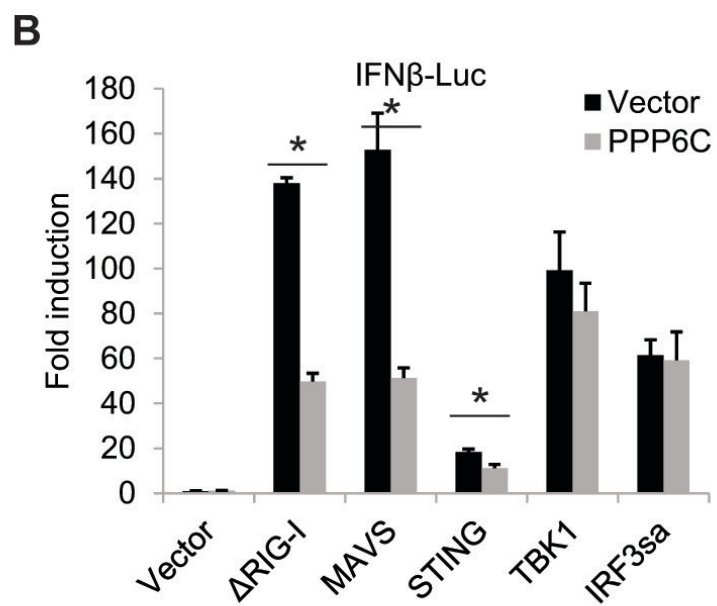

Supplement: FIG S4 [file mBio.01728-20-sf004.pdf]
