# Supplementary material for: PPP6C Negatively Regulates STING-Dependent Innate Immune Responses
Source: mBio. 2020 Aug 4;11(4):e01728-20. doi: 10.1128/mBio.01728-20 (PMC7407089; doi:10.1128/mBio.01728-20)
Supplement: TABLE S1 [file mBio.01728-20-st001.pdf]

Table S1. Human Interferons & Receptors PCR array in EA.hy926 cells

		2^(-Ava.(Delta(Ct))				Fold changes	
	Gene name	siNS dsDNA 0h	siPPP6C dsDNA 0h	siNS dsDNA 4h	siPPP6C dsDNA 4h	NS-4h /NS-0h	PPP6C-4h /NS-4h
1	ADAR adenosine deaminase RNA specific	1.10E-02	1.27E-02	1.93E-02	2.25E-02	1.75	1.17
2	CNTFR ciliary nerotrophic factor receptor	2.75E-06	2.93E-06	5.31E-06	7.86E-06	1.93	1.48
3	CRLF2 cytokine receptor like factor 2	1.63E-05	1.68E-05	9.79E-05	8.21E-05	5.99	0.84
4	CSF2RA colony stimulating factor 2 receptor alpha subunit	1.21E-06	8.65E-07	8.98E-07	2.26E-06	0.74	2.52
5	CSF3R colony stimulating factor 3 receptor	8.82E-07	9.19E-07	1.05E-06	1.24E-06	1.19	1.18
6	CXCL10 C-X-C motif chemokine ligand 10	3.52E-06	3.13E-06	9.21E-04	1.87E-03	261.60	2.03
7	EBI3 Epstein-Barr virus induced 3	9.07E-04	1.03E-03	9.32E-04	1.03E-03	1.03	1.11
8	F3 coagulation factor III, tissue factor	2.91E-05	3.81E-05	6.61E-05	1.00E-04	2.27	1.52
9	IFI16 interferon gamma inducible protein 16	1.15E-02	1.29E-02	2.79E-02	3.37E-02	2.44	1.21
10	IFI27 interferon alpha inducible protein 27	1.23E-02	1.50E-02	2.55E-02	3.54E-02	2.08	1.39
11	IFI30 IFI30 lysosomal thiol reductase	5.61E-04	6.00E-04	2.81E-03	4.13E-03	5.00	1.47
12	IFI35 interferon induced protein 35	2.42E-03	2.88E-03	6.06E-03	8.50E-03	2.50	1.40
13	IFI44 interferon induced protein 44	5.12E-03	6.45E-03	3.72E-02	4.21E-02	7.25	1.13
14	IFI44L interferon induced protein 44 like	3.64E-04	5.38E-04	1.83E-03	2.30E-03	5.04	1.26
15	IFI6 interferon alpha induced protein 6	9.44E-04	9.26E-04	4.22E-03	5.20E-03	4.47	1.23
16	IFIH1 interferon induced with helicase C domain	1.50E-03	1.94E-03	4.81E-02	5.46E-02	32.11	1.14
17	IFIT1 interferon induced protein with tetratricopeptide repeats 1	7.99E-03	1.01E-02	4.13E-01	4.72E-01	51.66	1.14
18	IFIT2 interferon induced protein with tetratricopeptide repeats 2	2.38E-03	2.93E-03	3.09E-01	3.82E-01	129.58	1.24
19	IFIT3 interferon induced protein with tetratricopeptide repeats 3	6.79E-03	8.05E-03	2.72E-01	3.31E-01	40.09	1.22
20	IFITM1 interferon induced transmembrane protein 1	3.68E-03	3.70E-03	9.09E-03	1.24E-02	2.47	1.37
21	IFITM2 interferon induced transmembrane protein 2	1.85E-02	1.86E-02	2.22E-02	2.35E-02	1.20	1.06
22	IFNA1 interferon alpha 1	2.47E-05	1.85E-05	3.00E-05	3.66E-05	1.21	1.22
23	IFNA14 interferon alpha 14	2.58E-07	2.66E-07	5.02E-07	3.66E-07	1.95	0.73
24	IFNA16 interferon alpha 16	4.51E-06	3.97E-06	3.71E-06	5.45E-06	0.82	1.47
25	IFNA2 interferon alpha 2	1.25E-05	9.89E-06	1.53E-05	3.27E-05	1.22	2.14
26	IFNA21 interferon alpha 21	1.40E-06	1.39E-06	6.04E-06	1.32E-05	4.32	2.18
27	IFNA4 interferon alpha 4	1.38E-05	1.35E-05	2.93E-05	6.72E-05	2.12	2.29
28	IFNA5 interferon alpha 5	1.68E-05	1.72E-05	2.53E-05	3.30E-05	1.51	1.30
29	IFNA6 interferon alpha 6	4.22E-05	4.34E-05	5.33E-05	8.16E-05	1.26	1.53
30	IFNA7 interferon alpha 7	5.16E-06	5.16E-06	7.85E-06	1.41E-05	1.52	1.79
31	IFNA8 interferon alpha 8	5.39E-06	6.28E-06	9.94E-06	2.17E-05	1.85	2.18
32	IFNAR1 interferon alpha and beta receptor subunit 1	3.68E-03	3.01E-03	3.28E-03	3.31E-03	0.89	1.01
33	IFNAR2 interferon alpha and beta receptor subunit 2	2.58E-03	2.80E-03	3.14E-03	3.26E-03	1.22	1.04
34	IFNBI interferon beta 1	1.58E-05	2.09E-05	1.98E-02	4.18E-02	1250.65	2.12
35	IFNE interferon epsilon	1.37E-04	1.03E-04	8.82E-05	6.95E-05	0.64	0.79
36	IFNG interferon gamma	2.19E-05	2.38E-05	2.15E-05	3.36E-05	0.98	1.56
37	IFNGR1 interferon gamma receptor 1	1.32E-03	1.17E-03	2.01E-03	1.77E-03	1.52	0.88
38	IFNGR2 interferon gamma receptor 2	2.98E-03	3.26E-03	2.96E-03	3.18E-03	0.99	1.07
39	IFNK interferon kappa	4.92E-05	4.98E-05	6.43E-05	6.57E-05	1.31	1.02
40	IFNW1 interferon omega 1	6.99E-07	4.73E-07	1.28E-05	4.58E-05	18.35	3.57
41	IFRD1 interferon related developmental regulator 1	2.79E-03	2.76E-03	3.27E-03	3.07E-03	1.17	0.94
42	ILRD2 interferon related developmental regulator 2	3.27E-03	3.35E-03	3.32E-03	3.16E-03	1.01	0.95
43	IL10RA interleukin 10 receptor subunit alpha	4.35E-06	6.29E-06	5.61E-06	7.97E-06	1.29	1.42
44	IL10RB interleukin 10 receptor subunit beta	2.38E-03	2.71E-03	1.99E-03	2.46E-03	0.84	1.24
45	IL11RA interleukin 11 receptor subunit alpha	1.01E-04	1.20E-04	1.11E-04	1.42E-04	1.10	1.28
46	IL12B interleukin 12B	5.34E-06	4.68E-06	5.20E-06	5.61E-06	0.97	1.08
47	IL13RA1 interleukin 13 receptor subunit alpha 1	1.98E-02	2.01E-02	2.00E-02	1.99E-02	1.01	0.99
48	IL15 interleukin 15	4.98E-04	5.36E-04	2.05E-03	1.86E-03	4.11	0.91
49	IL20RA interleukin 20 receptor subunit alpha	7.44E-05	8.84E-05	8.84E-05	1.04E-04	1.19	1.18
50	IL20RB interleukin 20 receptor subunit beta	8.28E-05	9.31E-05	8.86E-05	1.10E-04	1.07	1.24
51	IL21R interleukin 21 receptor	1.89E-06	1.56E-06	1.44E-06	1.19E-06	0.77	0.82
52	IL22RA2 interleukin 22 receptor subunit alpha	5.40E-06	7.04E-06	5.01E-06	8.30E-06	0.93	1.66

53	IL28A interferon lambda 2	2.38E-06	2.59E-06	1.36E-03	3.04E-03	570.46	2.24
54	IL28RA interferon lambda receptor 1	2.72E-04	3.24E-04	3.15E-04	4.21E-04	1.16	1.34
55	IL29 interferon lambda 1	2.56E-06	5.30E-06	2.15E-03	3.74E-03	838.86	1.74
56	IL2RB interleukin 2 receptor subunit beta	3.89E-06	3.92E-06	3.85E-06	4.32E-06	0.99	1.12
57	IL2RG interleukin 2 receptor subunit gamma	1.76E-06	2.34E-06	3.02E-06	3.68E-06	1.72	1.22
58	IL31RA interleukin 31 receptor A	1.36E-03	1.60E-03	1.02E-03	1.27E-03	0.75	1.25
59	IL3RA interleukin 3 receptor subunit alpha	7.34E-05	1.05E-04	8.48E-05	1.20E-04	1.16	1.42
60	IL4R interleukin 4 receptor	3.16E-03	3.12E-03	1.28E-03	1.40E-03	0.40	1.09
61	IL5RA interleukin 5 receptor subunit alpha	1.04E-06	6.95E-07	1.29E-06	1.84E-06	1.25	1.42
62	IL6 interleukin 6	1.76E-03	1.87E-03	1.08E-02	1.14E-02	6.11	1.06
63	IL6R interleukin 6 receptor	3.47E-04	6.26E-04	1.82E-04	3.50E-04	0.53	1.92
64	IL7R interleukin 7 receptor	1.54E-03	1.32E-03	8.71E-03	7.09E-03	5.65	0.81
65	IL9R interleukin 9 receptor	1.29E-06	1.45E-06	2.16E-06	4.49E-06	1.67	2.08
66	IRF1 interferon regulatory factor 1	8.17E-04	7.36E-04	2.10E-02	2.72E-02	25.63	1.30
67	IRF2 interferon regulatory factor 2	2.18E-03	2.53E-03	6.41E-03	8.12E-03	2.94	1.27
68	IRF2BP1 interferon regulatory factor 2 binding protein 1	1.81E-05	1.87E-05	2.08E-05	2.12E-05	1.15	1.02
69	IRF3 interferon regulatory factor 3	3.42E-03	3.87E-03	3.90E-03	4.38E-03	1.14	1.12
70	IRF4 interferon regulatory factor 4	4.00E-06	3.09E-06	3.29E-06	3.77E-06	0.82	1.15
71	IRF5 interferon regulatory factor 5	1.16E-05	1.06E-05	1.67E-05	1.87E-05	1.44	1.12
72	IRF6 interferon regulatory factor 6	6.08E-05	6.83E-05	7.02E-05	8.39E-05	1.15	1.20
73	IRF7 interferon regulatory factor 7	9.68E-04	9.84E-04	5.10E-03	6.11E-03	5.27	1.20
74	IRF8 interferon regulatory factor 8	1.92E-07	3.05E-07	3.91E-08	5.25E-07	0.20	13.45
75	IRGM immunity related GTPase M	1.51E-05	1.55E-05	1.48E-05	1.93E-05	0.98	1.31
76	ISG15 ISG15 ubiquitin like modifier	2.56E-03	2.82E-03	3.68E-02	4.06E-02	14.38	1.10
77	LEPR leptin receptor	1.66E-04	1.60E-04	1.77E-04	1.84E-04	1.07	1.04
78	MPL MPL proto-oncogene, thrombopoietin receptor	1.22E-05	1.57E-05	1.03E-05	1.56E-05	0.85	1.51
79	MX1 MX dynamic like GTPase 1	2.12E-04	2.54E-04	3.70E-04	5.16E-04	1.75	1.39
80	OAS1 2'-5'-oligoadenylate synthetase 1	6.27E-03	8.36E-03	5.17E-02	6.97E-02	8.26	1.35
81	PSME1 proteasome activator subunit 1	1.73E-02	1.85E-02	2.76E-02	3.10E-02	1.59	1.12
82	PYHIN1 pyrin and HIN domain family member 1	2.15E-07	1.59E-07	1.22E-07	1.25E-07	0.56	1.03
83	SP110 SP110 nuclear body protein	2.84E-03	3.50E-03	8.30E-03	1.11E-02	2.92	1.33
84	TTN titin	6.26E-06	8.37E-06	4.89E-06	6.34E-06	0.78	1.30
